# Microalgae *Pediastrum boryanum* enhances growth and modulates physiology and transcriptome in Nile tilapia

**DOI:** 10.1038/s41598-025-07498-1

**Published:** 2025-07-01

**Authors:** Raghad E. Soliman, Ahmed Ateya, Mohamed Abou-El-Atta, Wasseem Emam, Mohamed M. Abdel-Rahim, Ashraf I. G. Elhetawy, Ahmed E. Elshafey, Radi A. Mohamed

**Affiliations:** 1 Department of Aquaculture, Faculty of Aquatic and Fisheries Sciences, Kafrelsheikh University, Kafrelsheikh, Egypt; 2 Department of Development of Animal Wealth, Faculty of Veterinary Medicine, Mansoura University, Mansoura, 35516 Egypt; 3 Department of Fish Health and Management, Central Laboratory for Aquaculture Research, Abo-Hammad, Egypt; 4 Ethical Seafood Research, Glasgow, UK; 5 Aquaculture Division, National Institute of Oceanography and Fisheries, NIOF, Cairo, Egypt; 6 Key Laboratory of Freshwater Aquatic Genetic Resources, Ministry of Agriculture and Rural Affairs, Shanghai Ocean University, Shanghai, 201306 China; 7 Key Laboratory of Exploration and Utilization of Aquatic Genetic Resources, Ministry of Education, Shanghai Ocean University, Shanghai, 201306 China

**Keywords:** Microalgae, *Pediastrum boryanum*, Nile tilapia, Immunity, Transcriptomic response, Physiology, Zoology

## Abstract

The study aimed to explore the impact of dietary *Pediastrum boryanum* (PB) on growth performance, immune-physiological and transcriptome responses of Nile tilapia (*Oreochromis niloticus*). A 90-day trial using 1800 fish (17.00 ± 1.722 g) was allocated at random into 4 groups, three replicates each. The 4 groups received PB at 0 (G1), 1 (G2), 2 (G3), and 4 (G4) g/kg diet, respectively. The fish in Group G3 displayed a significant (*p* < 0.05) increase in final body weight, weight gain, specific growth rate, and final length when compared to the control group. In addition, red blood cell counts (RBCs), and neutrophils were significantly (*p* < 0.05) higher in G3. The groups’ biochemical parameters did not significantly differ from one another; nevertheless, significant (*p* < 0.05) variations in immunoglobulin M (IgM), phagocytic activity, and the values of the phagocytic index. The antioxidative activity of superoxide dismutase (SOD) and catalase differed significantly (*p* < 0.05) between groups, with the highest values seen in group G3. Moreover, there was a significant (*p* < 0.05) upgrade in the expression levels of *GH*, *IGF-1*, *SOD*, *catalase*, *IL-6*, *IL-8*, and *PGAM2* genes in group G3. Additionally, G3 had the most typical liver structure, significantly (*p* < 0.05) increased villi length and width, and a thicker lamina propria in the intestine. In conclusion, dietary supplementation of PB boosted growth performance, blood and serum profile, morphometric evaluation of the liver and intestine, and expression of genes related to growth, antioxidant capacity, immunity, and energy metabolism in Nile tilapia.

## Introduction

In tropical and subtropical aquaculture, the Nile tilapia, *Oreochromis niloticus* is one of the most important fish species^1^. It provides significant sources of revenue and animal protein throughout the world^2,3^. The consumption of tilapia has risen globally as it has emerged as aquaculture’s brightest star and is also sometimes referred to as “aquatic chicken“^4^. Tilapia can develop and reproduce in a variety of environmental circumstances and can withstand handling-induced stress^5,6^. Tilapia is known for its fast growth, ability to adapt to various environments including high stocking densities, strong disease resistance, and tolerance for adverse water quality. Additionally, the use of antibiotics and chemicals is minimal in commercial farming, production costs are low, it has white flesh, and its meat can be processed into many value-added products. It also possesses good market demand and is well-accepted by consumers^6,7^.

One of the most globally important industries is aquacultures, which provides necessary food for the growing world population and has an important role in providing high quality and cheap animal protein^8^. Fast developments have been made in Egypt in the aquaculture sector in the last years and showed the biggest growth among all fisheries-related activity in the country^9^. So, aquaculture is considered a suitable option to reduce the current gap between fish consumption and production in Egypt^10^.

Despite the success of aquaculture in Egypt, it still faces some problems, such as incorrect production of seed, and the lack of proper breeding and feeding programs for fish and fingerlings, high price of feed, incorrect use of water and large quantities wasted due to misuse, contamination of water with ammonia and harmful mute substances that cause the death of fish, excessive use of Nile water in fish farms, poor quality of feed that is used to feed fish, poor quality seed that is infected with diseases, and problems of oxygen deficiency^11–13^. To overcome these problems, various algae, algae extracts and phytobiotics attract attention as possible solutions for cultured fish^14–16^. *Pediastrum* is recognized as an important supply of bioactive substances because of its capacity to generate an extensive range of secondary metabolites, which exhibit wide-ranging biological activities against both human and fish pathogens^17^.

Several studies have focused on microalgae in recent years. The study of culture conditions or food availability encouraging the synthesis of various metabolites, like polyunsaturated fatty acids, phenolic compounds, and polysaccharides, has made significant strides in biotechnology^18–20^. Experimental models, exhibited both in vitro and in vivo the pharmacological properties of microalgae, including their effectiveness in preventing diabetes, their anti-cancer capabilities, as well as their anti-pain and anti-inflammatory effects^21–23^.

Spirulina, chlorella, and other microalgae have demonstrated anti-inflammatory and antioxidant capabilities^24,25^. Nowadays, an intriguing area of research in aquaculture is the consumption of fish that has been treated with microalgae as a source of protein, natural antioxidants, and immunological preservatives. Microalgae are rich in polyunsaturated fatty acids, amino acids, polysaccharides, minerals, vitamins, and various colorants such as phycobiliproteins, carotenoids, and chlorophylls, among other chemicals with additional value^26,27^.

Even though there are hundreds of microalgae species, only a few of their medicinal potential have been fully examined. Therefore, the current study had an emphasis on *Pediastrum boryanum* (PB) in this context. PB is a potentially abundant source of antioxidants, particularly phenolic compounds^28^. Prior research has demonstrated that this microalga is an important source of antioxidant chemicals and anti-inflammatory^29^. Very little is known about PB in the field of aquaculture; hence this search was aimed to investigate the impact of dietary supplementation of *Pediastrum boryanum* (PB) on feed utilization, performance, water quality, immune response, antioxidant activity, transcriptomic response, hepatopancreas and intestinal tract histomorphology in Nile tilapia.

## Materials and methods

### Ethical approval

The experimental procedures for fish in this study adhered to Egyptian legislation on ethics in fish use and handling and were approved by the Committee of Aquatic Animal Care and Use in Research at the Faculty of Aquatic and Fisheries Sciences, Kafrelsheikh University, Egypt (approval number: IAACUC-KSU-2-2018). The study is reported in accordance with ARRIVE guidelines for animal research and all experiments were performed in accordance with relevant guidelines and regulations.

### Algae preparation and experimental design

*Pediastrum boryanum* (PB) was identified, collected, cultivated, dried, ground, and sieved at the Algae Technology Unit of the National Research Center in Dokki, Egypt. The microalgae were meticulously processed to yield a refined product. The required amount of PB for this trial was purchased in fully and ready to use powder form from the Algae Technology Unit, the National Research Center, Dokki, Egypt. The experiment was subsequently conducted at New Hope Fish Farm in Kafr El-Sheikh, Egypt (31°25′53.8′′ N 30°59′05.3′′ E). One thousand and eight hundred healthy monosex Nile tilapia (*O. niloticus*) were procured according to biosecurity protocols. The fish’s health status was evaluated by monitoring clinical signs during a two-week acclimatization period. The fish were raised in twelve large cement ponds, each holding 150 fish (3 × 8 m, 24 m^2^) and having a depth of 90 cm. These ponds represented the four experimental treatments, each with three replicates.

### Experimental treatments

Fish (initial weight: 17.00 ± 1.722 g) were fed four treatment diets in form of triplicates for 90 days. Where G1 is the control group and fish feed basal diet only (30% protein, produced by New Hope Aquatic, Gamasa, Dakahlya, Egypt), G2: the fish feed diet supplemented with 1 g PB powder /Kg, G3: the fish feed diet supplemented with 2 g PB powder /Kg, and G4: the fish feed diet supplemented with 4 g PB powder /Kg. Supplemented diets (Table 1) were prepared by spraying PB powder (dissolved in nutria-B gel 20 ml/Kg) uniformly on the commercial feed. For the control group (G1), the feed was sprayed and mixed with the same amount of gel. The diet was mixed properly with PB several times to ensure equal and even distribution of the PB amount on the diet. After that, the feed was dried at room temperature for 24 h and stored in the refrigerator until further use and chemical analysis. Feed was given out twice a day at 8:00 a.m. and 5:00 p.m. at a rate of 6% of the live body weight of the fish and decreasing based on changes in fish biomass that are recalculated every two weeks.

**Table 1:** Ingredients and chemical analysis of control, experimental diets and microalgae *Pediastrum boryanum* (PB).

Items	G1	G2	G3	G4	PB*
Ingredients	Kg				Gram
Brocken rice	137	137	137	137	PB powder 250
Wheat middling	80	80	80	80	
Soft rice whole powder	75.8	75.8	75.8	75.8	
Soft wheat bran	70	69	68	66	
Rice bran	30	30	30	30	
Soya oil	10	10	10	10	
Chicken meat meal (55% CP)	110	110	110	110	
Soybean meal (46% CP)	350	350	350	350	
Dried distillers grain solids (DDGS)	65	65	65	65	
Corn gluten meal (60% CP)	30	30	30	30	
Crushed corn grains	25	25	25	25	
Monocalcium phosphate	10	10	10	10	
Lysine (98.5%)	1.2	1.2	1.2	1.2	
DL-methionine (99%)	0.4	0.4	0.4	0.4	
Coline chloride (60%)	2	2	2	2	
Butylated hydroxytoluene (10%)	0.2	0.2	0.2	0.2	
Vitamin C (35%)	0.4	0.4	0.4	0.4	
^a^Mineral premix	2	2	2	2	
^b^Vitamin premix	1	1	1	1	
*Pediastrum boryanum* (PB)	0	1	2	4	
Chemical analysis	%				
Moisture (%)	9.8	10.84	10.38	10.33	11.72
Crude protein (%)	30.1	30.42	31.58	31.67	47.56
Crude lipid (%)	8.14	8.14	8.08	8	0.51
Crude fiber (%)	8.04	7.87	7.12	7.13	4.89
Ash (%)	7.67	7.5	7.58	7.61	53.17
Calcium (%)	0.89	0.86	0.83	0.81	9.98
Phosphorous (%)	1.23	1.22	1.24	1.25	1.97
Nitrogen-free extract**(%)	43.92	44.07	44.64	44.79	6.13
Gross energy (MJ/Kg diet)	1841.407	1842.75	1844.02	1842.36	1248.6

^a^Mineral premix (g/kg premix): manganese 7.5, magnesium 12, zinc 16, iron 42, cupper 2.5, iodine 0.5, selenium 0.15, cobalt 0.075, calcium carbonate carrier up to 1.

^b^Vitamin premix (g or IU/kg premix): VA 900,000, VD3 3,000,000, VE 80, VK3 6, VB1 9.6, VB2 12, VB6 15, VB12 0.02, VB3 40, VB5 36, folic acid 5.76.

The premixes are provided by New Hope, Singapore premix Pte Ltd.

*Pediastrum boryanum microalgae.

**Nitrogen-free extract = 100 − (% crude protein + % crude lipid + % ash+ % crude fiber).

### Chemical analyses of diets and *Pediastrum boryanum*

All the dry ingredients, including the experimental diets and PB, underwent analysis for moisture, protein, lipid, fiber, ash, calcium, and phosphorus following the method outlined in the^30^ guidelines. The amount of energy in each diet and *Pediastrum borianum* was determined using an adiabatic bomb calorimeter, in accordance with the^31^ standard as shown in Table 1.

### Growth performance and feed utilization

Using a suitable net, the fish were harvested from the concrete ponds. The fish were transported into a big plastic container (70 L). Tricaine methane sulfonate (MS222) from Ardent Laboratories in Redmond, Washington, USA (25 mg/L), was used to anesthetize 15 fish from each cement pond that were selected at random (45 fish per treatment) once the trial ended. After that, the whole remaining fish in each pond were harvested and counted for estimation of the survival rate and total biomass. The ultimate body weight of each fish was ascertained by weighing it separately using a portable balance (Adam Equipment, China). To obtain the growth assessment variables, the following formulas were applied^32^: Body weight gain (BWG) = final body weight (FBW) − initial body weight (IBW). Feed intake (FI) = the amount of feed given to fish during the experimental period. Feed conversion ratio (FCR) = (FI) (g)/BWG (g). Specific growth rate (SGR % / day) = 100 × (ln FBW − ln IBW)/t. Condition factor (K) = 100 x (FBW (g)/cubic length cm). Survival rate (SR%) = [End of fish count/beginning of fish count] x 100. Here is the duration of the experiment in days.

### Blood sampling

Blood was drawn using syringes coated with EDTA anticoagulant attached to Nipro 27 gauge × 11/2-inch needles from the caudal veins of six fish representing each treatment. The collected blood was centrifuged (SIGMA 4–16 K refrigerated centrifuge, Sigma Laborzentrifugen, Germany) in plain tubes devoid of anticoagulants at 4 °C at 3000 rpm for 15 min for serum separation. After that, the serum was kept at – 20 °C until it was needed^33^.

### Hematology and blood biochemical analyses

Red blood cells (RBCs) were calculated using a hemocytometer and Natt–Herrik solution, Hemoglobin (Hb) concentration was ascertained using the cyanmethemoglobin method and Drabkin’s solution, and the microhematocrit technique worked for the determination of the packed cell volume (PCV)^34^.

As per^34^ the usual technique, it was used to count the white blood cells (WBCs), lymphocytes, neutrophils, and monocytes. According to^35^ studies of albumin, cholesterol, triglycerides, aspartate aminotransferase^36^ alanine aminotransferase (ALT), and total proteins (TP) were carried out. Commercial kits from Sigma-Aldrich, USA, were used to quantify serum TP at 540 nm. Serum albumin was assessed at 550 nm^37^ and the globulin concentration was computed using mathematics. Utilizing kits supplied by Bio-Diagnostic Co. (Dokki, Cairo, Egypt), total cholesterol, triglycerides were also measured at 540 nanometers using calorimetry. According to^38^ employed the colorimetric approach to evaluate the levels of creatinine. At 540 nm, the calorimetric assessment of AST and ALT activities was conducted with commercial kits that were readily available^35^.

### Immune and oxidative stress responses

Phagocytic activity and index were measured using prepared blood smears. The phagocytic activity was calculated by the following formula: PA = macrophages containing yeast/total number of macrophages ×100. According to^28^ PI = Number of phagocytized cells /Total number of phagocytic cells. Serum lysozyme activity and IgM were assayed using a microplate ELISA reader set at 450 nm, following the procedure from^39^. Superoxide dismutase (SOD), catalase (CAT), and malondialdehyde activities using an ELISA kit (Inova Biotechnology, China) at 450 nm, following the manufacturer’s instructions^40^.

### RNA extraction and quantitative real-time PCR

RNA extraction and cDNA synthesis were carried out using liver, spleen, and muscle tissue samples from Nile tilapia (6 fish/ treatment). After dissection of the fish and selection of the target organ (1 gram of liver and muscle and the whole spleen), the samples were shocked in liquid nitrogen. The samples were then stored at – 80 °C until analysis. RNA was extracted using TRIzol^®^ reagent and its content and purity were established using a Nanodrop Q5000 UV-Vis’s spectrophotometer and gel electrophoresis. cDNA was synthesized using the SensiFAST™ cDNA Synthesis Kit and stored at 4 °C.

Real-time PCR was performed using SYBR Green PCR Master Mix (2× SensiFASTTM SYBR, Bioline, Catalog No. Bio-98002) to quantify the mRNA levels of growth, immune, antioxidant, and energy markers. The amplified PCR product sizes and primer sequences for the genes under investigation are listed in Table 2. The housekeeping gene β-actin functioned as an internal control. 10 µl of 2× SensiFAST SYBR, 3 µl of cDNA, 5.4 µl of nuclease-free water, and 0.8 µl of each primer were added to each 20 µl reaction. The following were the conditions for PCR cycling: an eight-minute initial denaturation at 95 °C, followed by 40 cycles of denaturation for 15 s at 95 °C, annealing for 60 s at the temperatures specified in Table 2, and extension for 15 s at 72 °C. Following amplification, a melting curve analysis was carried out to verify the PCR product’s specificity. The relative expression of each gene was measured using the 2^−ΔΔCt^ method^41^. The results were then normalized to the housekeeping gene, β-actin, and compared to the control.

**Table 2:** Primers used for qRT-PCR analysis.

Gene	Source of isolation	Gene bank accession number	Nucleotide sequence of primers (5′–3′)	Annealing temp. (°C)	Size (bp)
*IL-6*	Spleen	XM_019350387.2	F: AAACCAATTCCTTCTGGCCCT R: TCACCTGAGAAGTCACCTGC	56	189
*IL-8*	Spleen	NM_001279704	F: CTGTGAAGGCATGGGTGTG R: ATCACTTTCTTCACCCAGGG	58	196
*CAT*	Liver	JF801726.1	F: TTTGCACGTTTACAGCCGTC R: CTGATGGCTCGAATAATGGTCC	60	129
*SOD*	Liver	JF801727.1	F: ACGTGACAACACAGGTTGCT R: AGTCCCGTTTGATTGCCTCC	58	144
*GH*	Liver	XM_003442542	F: TAATGGGAGAGGGA AGATGG R: CTCTGCGATGTAATTCAGGA	60	202
*IGF-I*	Liver	XM_003448059	F: TTGTCTGTGGAGAGCGAGGCTT R: CAGCTTTGGAAGCAGCACTCGT	62	90
*PGAM2*	Muscle	XM_003444358.5	F: GCTATCAAGGAGGCAGGCAT R: GACGCCAGGTACGAATCACA	58	130
*β-actin*	Spleen, liver, and muscle	XM_003443127	F: GTGCCCATCTACGAGGGTTA R: CTCCTTAATGTCACGCACGA	60	156

Interleukin-6 (IL-6), interleukin-8 (IL-8), superoxide dismutase (SOD), catalase (CAT), growth hormone (GH), insulin growth factor 1 (IGF-1), phosphoglycerate mutase 2 (PGAM2), and housekeeping (β-actin) gene.

### Liver and intestine histomorphology

Nine fish per treatment were used, and the liver and intestinal tissues were eliminated and preserved for three days in 10% neutral buffered formalin. Subsequently, the samples underwent dehydration, several washes in absolute alcohol, and paraffin embedding. 5 μm longitudinal slices were cut with a Leica RM 2145 rotary microtome (Leica Microsystems, Wentzler, Germany). The slices were then put on glass slides and stained with hematoxylin and eosin (H&E)^42^. Utilized was a Leica microscope modified to fit a Leica camera. Using ImageJ analysis software (National Institutes of Health, Bethesda, MD, USA), a histomorphometric investigation was carried out. The villus height (measured from the villus tip to the villus-crypt junction) and breadth (measured from the villus midpoint) were quantified using ImageJ analysis software.

### Water quality

Every day, multiparameter probe meter (Hanna Instruments, France- HI9829–03042-HANNA^®^insrruments, www.hannainst.com) was used in the center of each tank to measure parameters related to water quality, including temperature, pH, and dissolved oxygen (DO). Ammonia and nitrite levels were determined once every week using a spectrophotometer (Perkin Elmer lambda 25 UV/Vis, Canada) following the methods outlined in^43^.

### Statistical analysis

Using GraphPad Prism 6.01^®^ statistics software, a statistical analysis was carried out following the verification of the data for homogeneity and normality. One-way analysis of variance (ANOVA) and Tukey’s post hoc test were used to examine the data for any significant differences between the tested groups. Differences were deemed statistically significant when the *p*-value was less than 0.05. The results were expressed in mean and standard error of the mean (SEM).

## Results

### Growth performance and feed utilization

Growth performance parameters and feed utilization efficiency of Nile tilapia fed different dietary doses of PB are presented in Table 3. Final body weight, weight gain, feed intake, specific growth rate, and final length were significantly higher (*p* < 0.05) in fish fed diets supplemented with PB when compared with the control group (G1). The most favorable outcomes were noted in fish fed 2 g PB/kg (G3). Additionally, fish fed PB-supplemented diets demonstrated a significant reduction (*p* < 0.05) in the feed conversion ratio (FCR) compared to the control group, with the lowest FCR values recorded in fish fed 2 g PB/kg (G3). However, the condition factor remained unaffected by dietary PB supplementation (*p* > 0.05).

**Table 3:** Growth performance and feed utilization efficiency (mean ± SE) of fish fed experimental diets for 90 days.

Items	G1	G2	G3	G4	*P*-value
Initial body weight (g)	17.28 ± 1.641	17.00 ± 1.577	16.06 ± 2.338	17.67 ± 1.333	0.773
Final body weight (g)	165.9 ± 4.667^b^	167.1 ± 5.049^b^	192.6 ± 6.883^a^	151.9 ± 4.091^b^	0.000
Weight gain (g)	148.0 ± 4.667^b^	150.1 ± 5.049^b^	176.5 ± 6.883^a^	134.23 ± 4.091^b^	0.000
Feed intake (g)	223.8 ± 7.902	219.5 ± 9.937	238.6 ± 10.9	201.1 ± 6.697	0.054
Feed conversion ratio	1.511 ± 0.015^b^	1.459 ± 0.017^ab^	1.350 ± 0.018^a^	1.498 ± 0.021^b^	0.000
Specific growth rate (%/day)	2.475 ± 0.032^bc^	2.537 ± 0.032^b^	2.756 ± 0.040^a^	2.367 ± 0.031^c^	0.000
Final length (cm)	20.74 ± 0.194^ab^	21.09 ± 0.229^a^	21.79 ± 0.365^a^	19.69 ± 0.376^b^	0.001
Condition factor	1.858 ± 0.041	1.791 ± 0.080	1.864 ± 0.049	2.006 ± 0.089	0.176
Survival rate (%)	96.00 ± 0.924	97.60 ± 0.462	97.07 ± 0.705	95.73 ± 0.706	0.286

Means within the same row lack common superscripts are significantly different at *P* < 0.05.

### Hemato-biochemical profile

PB addition in the diet of Nile tilapia significantly influenced erythrogram parameters (Hb, RBCs, and PCV), with significantly higher values (*p* < 0.05) observed in fish fed PB-enriched diets compared to the control group, as shown in Table 4. However, the biochemical profile of fish fed PB-supplemented diets did not differ significantly (*p* > 0.05) from the control group (Table 5). Fish fed PB-enriched diets demonstrated elevated levels of total protein, albumin, and globulin, while exhibiting lower levels of cholesterol and glucose. The most pronounced effects on erythrogram parameters and biochemical markers were observed at a PB inclusion rate of 2 g/kg (G3).

**Table 4:** Hematological parameters (mean ± SE) of fish fed experimental diets for 90 days.

Items	G1	G2	G3	G4	*P*-value
RBCs (x106/mm³)	1.915 ± 0.055^b^	2.200 ± 0.098^a^	2.290 ± 0.006^a^	2.165 ± 0.026^ab^	0.010
Hb (g/dL)	8.745 ± 0.267^b^	9.450 ± 0.144^ab^	9.790 ± 0.006^a^	10.01 ± 0.113^a^	0.003
PCV (%)	31.50 ± 0.866^a^	27.50 ± 0.289^b^	27.00 ± 0.577^b^	28.00 ± 0.578^b^	0.003
WBCs (x10³/mm³)	110.60 ± 2.335^a^	108.9 ± 0.335^a^	104.6 ± 1.385^b^	108.2 ± 1.030^ab^	0.006
Lymphocytes (%)	26.00 ± 0.578^c^	32.50 ± 0.289^b^	30.00 ± 0.577^b^	34.50 ± 0.866^a^	0.000
Neutrophils (%)	56.00 ± 2.100^b^	56.00 ± 2.200^b^	61.50 ± 1.500^a^	60.50 ± 0.500^a^	0.005
Monocytes (%)	5.700 ± 0.500	7.350 ± 0.650	6.300 ± 0.200	6.450 ± 0.850	0.055

Means within the same row lack common superscripts are significantly different at *P* < 0.05.

Red blood cells (RBCs), hemoglobin (Hb), packed cell volume (PCV), white blood cells (WBCs).

**Table 5:** Biochemical parameters (mean ± SE) of fish fed experimental diets for 90 days.

Items	G1	G2	G3	G4	*P*-value
TP (g/dL)	3.750 ± 0.550	3.600 ± 0.300	3.850 ± 0.150	3.650 ± 0.050	0.788
Albumin (g/dL)	1.500 ± 0.231	1.550 ± 0.144	1.650 ± 0.029	1.667 ± 0.176	0.532
Globulins (g/dL)	1.850 ± 0.259	1.950 ± 0.260	2.300 ± 0.058	2.183 ± 0.148	0.415
Triglyceride (mg/dL)	214.3 ± 6.692	217.7 ± 6.489	207.7 ± 6.227	219.7 ± 3.756	0.532
Cholesterol (g/dL)	166.5 ± 2.598	158.5 ± 5.635	158.5 ± 5.393	160.0 ± 1.155	0.507
Creatinine (mg/dL)	0.321 ± 0.012	0.342 ± 0.011	0.374 ± 0.037	0.400 ± 0.055	0.344
ALT (U/l)	18.50 ± 2.021	19.50 ± 0.289	21.50 ± 0.866	23.00 ± 0.577	0.089
AST (U/l)	162.7 ± 4.978	174.3 ± 10.91	169.0 ± 6.658	163.7 ± 3.667	0.647
Glucose (mg/dL)	59.00 ± 2.309	56.50 ± 2.363	56.00 ± 2.309	56.83 ± 1.878	0.788

Means within the same row lack common superscripts are significantly different at *P* < 0.05.

Alanine aminotransferase (ALT), aspartate aminotransferase (AST), total protein (TP).

### Immune assay

**Fig. 1:**
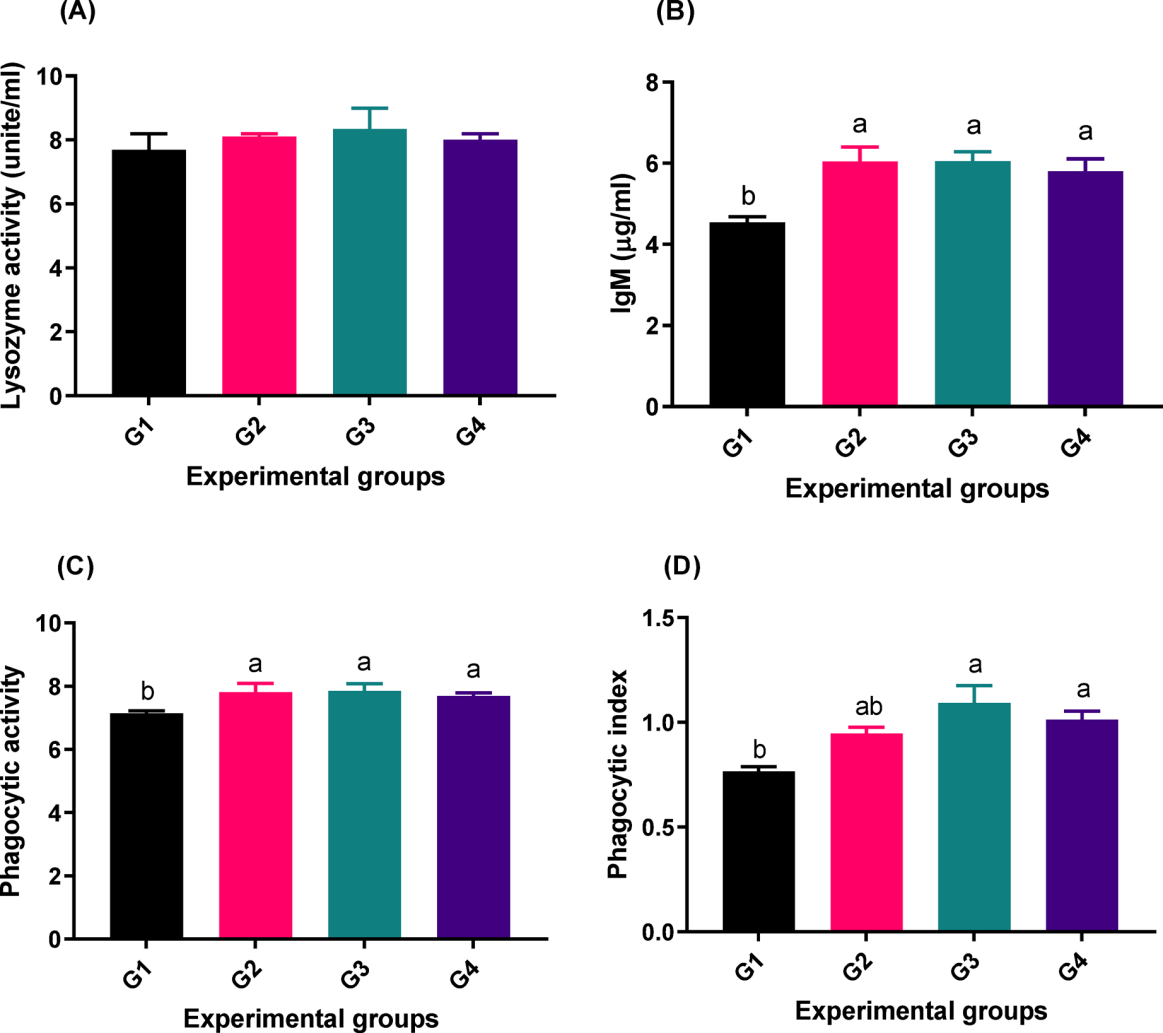
Immune response [lysozyme activity (**A**), Immunoglobulin M (IgM) (**B**), Phagocytic activity, (**C**) and phagocytic index (**D**)] of fish fed different experimental diets. The control group, G1, received a basal diet, while G2, G3, and G4 received diets supplemented with 1 g, 2 g, and 4 g of PB powder per kilogram of feed, respectively for 90 days. The columns (mean ± SEM) with different letters are significantly different (*P* < 0.05).

Results in Fig. 1 display significant differences (*p* < 0.05) in immunoglobulin M (IgM) (Fig. 1; B), phagocytic activity (Fig. 1; C), and phagocytic index (Fig. 1; D) values among the groups. On the other hand, there were no significant differences (*p* > 0.05) in lysozyme activity (Figure.1; A) between all treatments. The groups (G2, G3, and G4) where fish were given different doses of PB had the greatest value in all immune parameters (especially G3) compared to the control.

### Oxidative stress activities

**Fig. 2:**
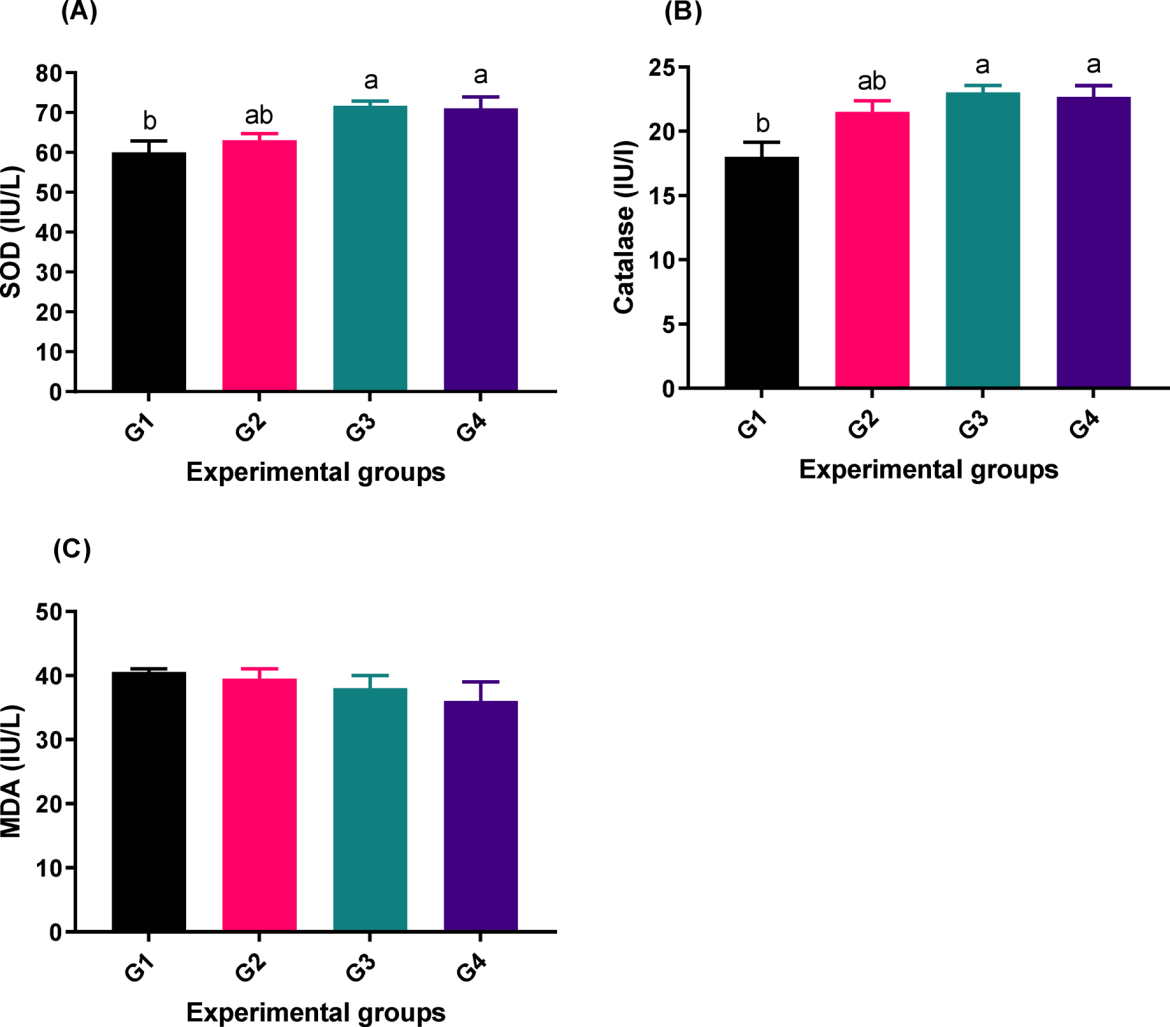
Oxidative parameters [SOD (**A**), catalase (**B**), and MDA (**C**)] of fish fed experimental diets. The control group, G1, received a basal diet, while G2, G3, and G4 received diets supplemented with 1 g, 2 g, and 4 g of PB powder per kilogram of feed, respectively for 90 days. The columns (mean ± SEM) with different letters are significantly different (*P* < 0.05). SOD = Superoxide dismutase, MDA = malonaldehyde.

As indicated in Fig. 2, there was a significant difference in superoxide dismutase (SOD) (Fig. 2; A) and catalase (Fig. 2; B) among the groups (*p*-values are 0.0161 and 0.0151, respectively). The highest values were found in the group fed with 2 g PB/kg (G3). No significant difference was noted in malonaldehyde (*p* > 0.05) among the groups (Fig. 2; C), and the lowest value was seen in the groups that received PB supplements (G2, G3, and G4) compared to the control group.

### Gene expression assay

**Fig. 3:**
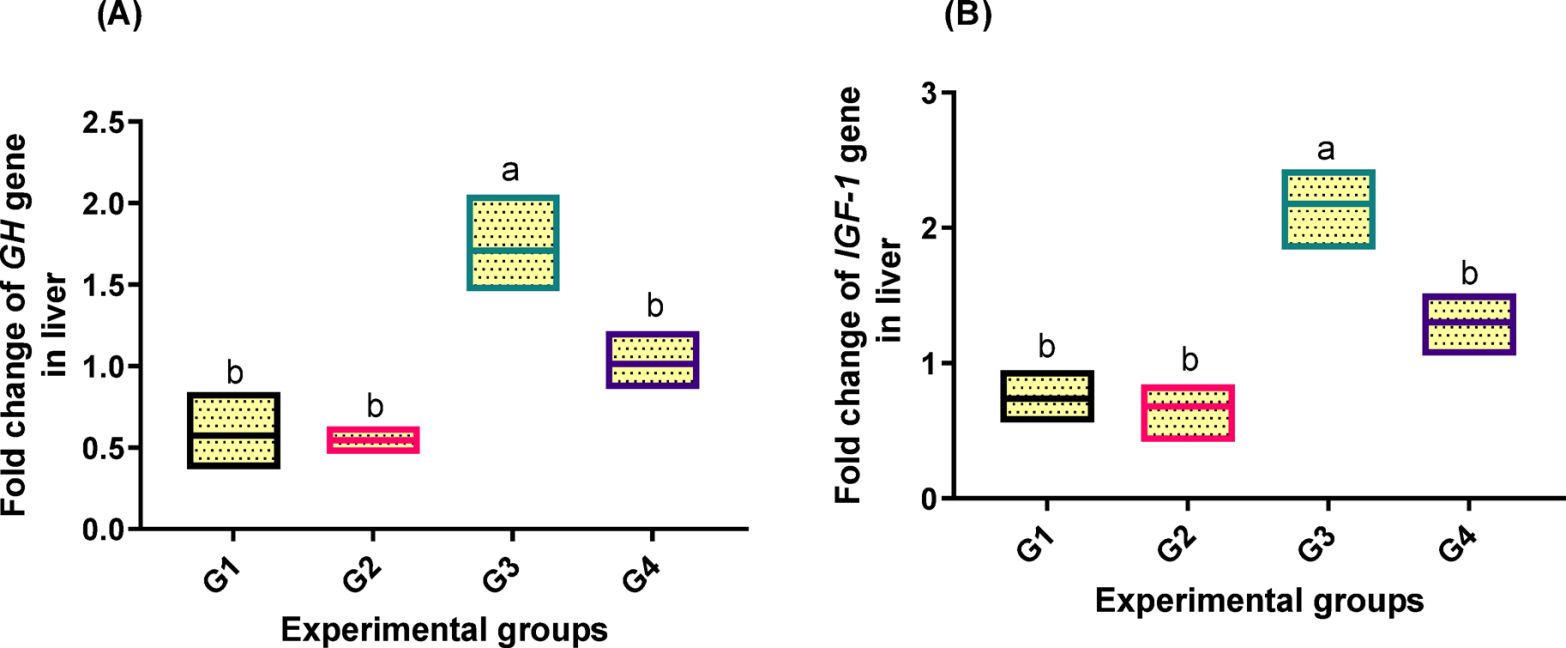
Transcriptomic profiles of genes [(**A**) growth hormone (GH) and (**B**) insulin growth factor 1 (IGF-1)] in liver tissue related to growth of fish fed experimental diets. The control group, G1, received a basal diet, while G2, G3, and G4 received diets supplemented with 1 g, 2 g, and 4 g of PB powder per kilogram of feed, respectively for 90 days. Floating bars show the minimum and maximum values also the middle line shows the mean value, and the different letters above each Floating bars showed significantly different (*P* < 0.05) among the means, which are presented as mean ± SEM.

Fish fed varying amounts of PB showed significantly different levels of growth-related genes in their livers (*p* < 0.05) in relation to the other groups. The fish received 2 g/kg PB (G3) had the highest upregulation of *GH* and *IGF-1* (Fig. 3; A and B) gene expression, with 1.709 and 2.178-fold increases, respectively.

**Fig. 4:**
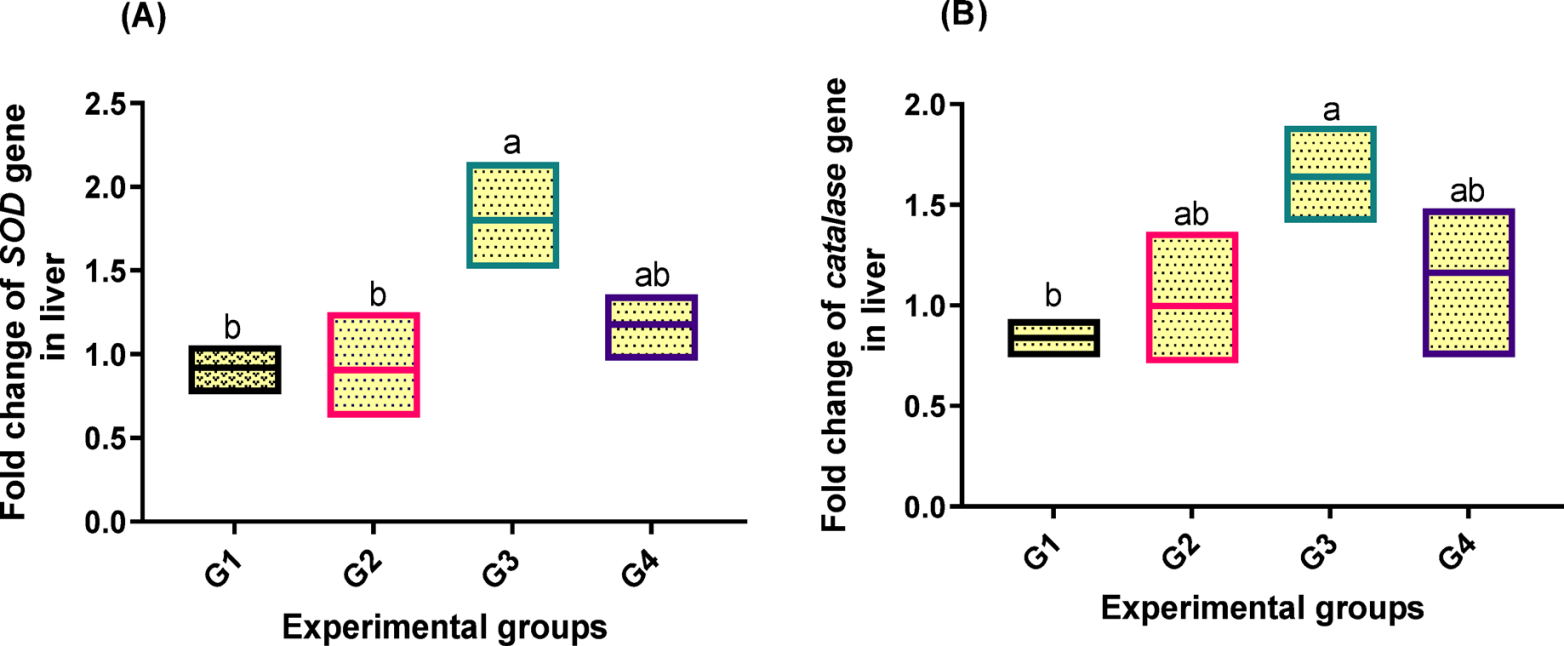
Transcriptomic profiles of genes [(**A**): superoxide dismutase (SOD) and (**B**) catalase] in liver tissue related to antioxidant capacity of fish fed experimental diets. The control group, G1, received a basal diet, while G2, G3, and G4 received diets supplemented with 1 g, 2 g, and 4 g of PB powder per kilogram of feed, respectively for 90 days. Floating bars show the minimum and maximum values also the middle line shows the mean value, and the different letters above each Floating bars showed significantly different (*P* < 0.05) among the means, which are presented as mean ± SEM.

Antioxidant capacity-related gene expressions in the liver revealed a significant difference (*p* < 0.05) between *SOD* and *CAT* levels (Fig. 4; A and B). The highest levels of upregulation (1.800, 1.639-fold increase, respectively) were noted in fish fed 2 g/kg PB in the diet (G3) in relation to the other groups.

**Fig. 5:**
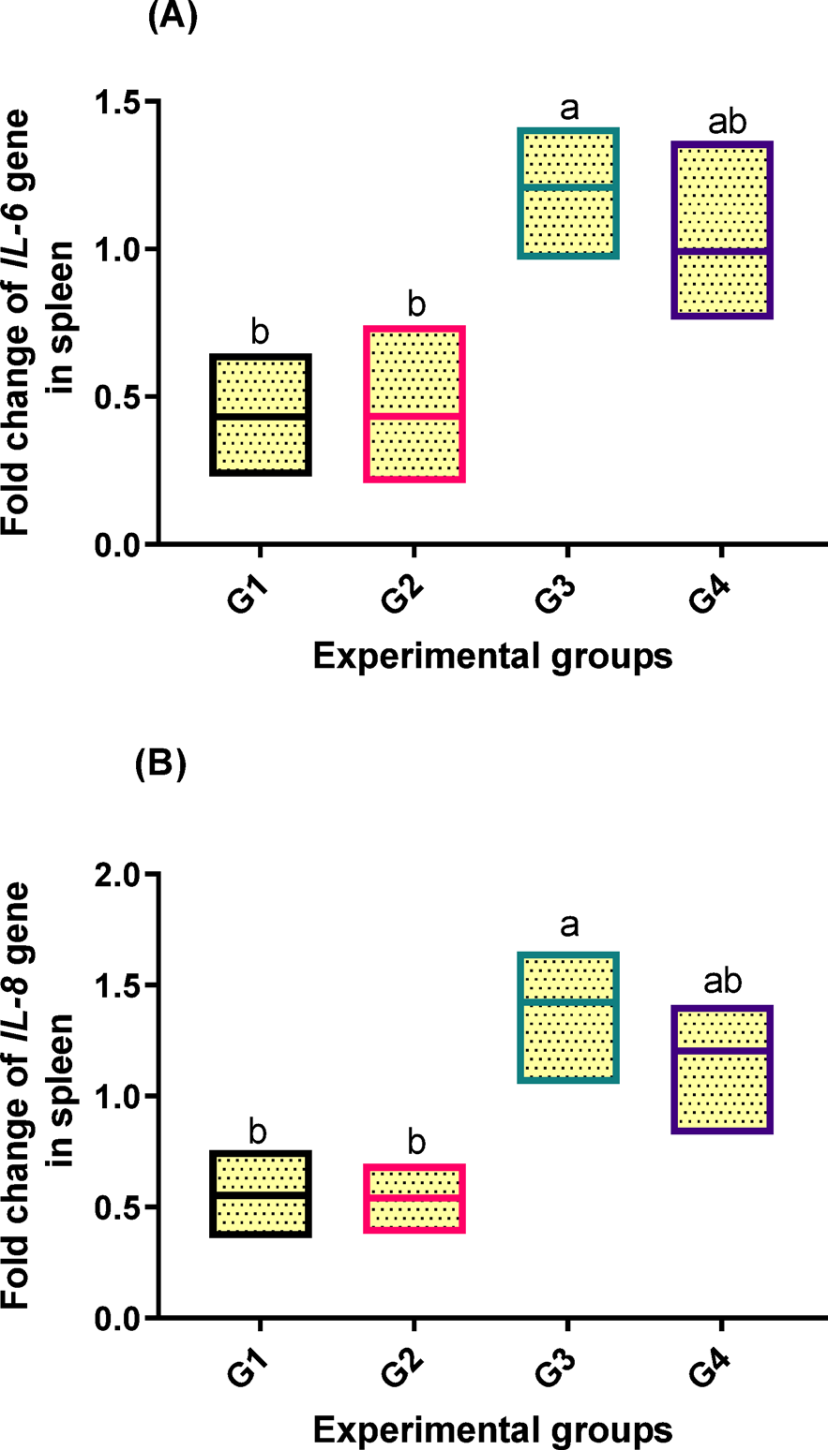
Transcriptomic profiles of genes [(**A**) interleukin-6 (IL-6) and (**B**) interleukin-8 (IL-8)] in spleen tissue related to immune response of fish fed experimental diets. The control group, G1, received a basal diet, while G2, G3, and G4 received diets supplemented with 1 g, 2 g, and 4 g of PB powder per kilogram of feed, respectively for 90 days. Floating bars show the minimum and maximum values also the middle line shows the mean value, and the different letters above each Floating bars showed significantly different (*P* < 0.05) among the means, which are presented as mean ± SEM.

Fish fed varying doses of dietary PB showed significant (*p* < 0.05) differences in the levels of immune response-related gene expressions in their spleens. Figure 5; A and B showed that fish fed 2 g/kg PB in the diet (G3) displayed the highest upregulation levels of *IL-6* and *IL-8* genes (1.209 and 1.423-fold increase, respectively).

**Fig. 6:**
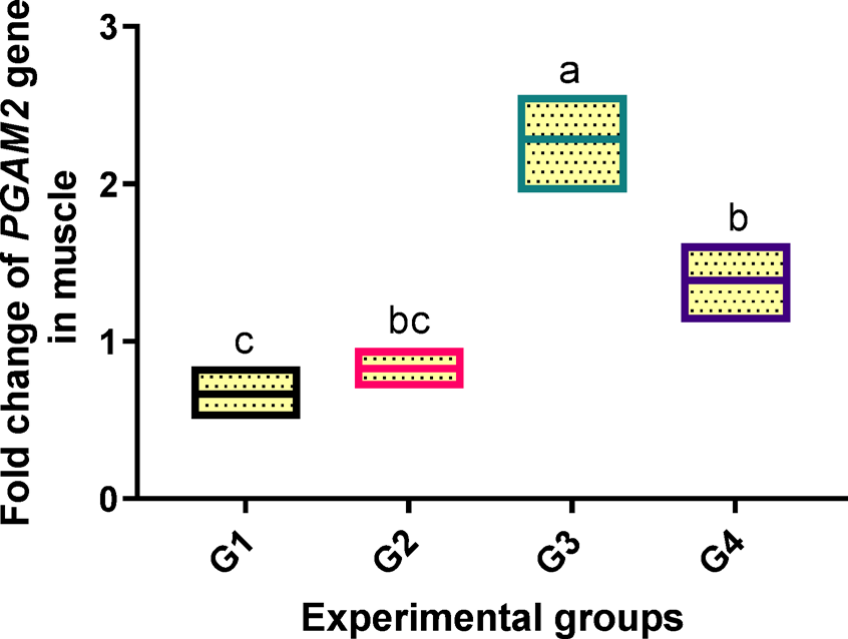
Transcriptomic profiles of phosphoglycerate mutase 2 (PGAM2) gene in muscle tissue related to energy production of fish fed experimental diets. The control group, G1, received a basal diet, while G2, G3, and G4 received diets supplemented with 1 g, 2 g, and 4 g of PB powder per kilogram of feed, respectively for 90 days. Floating bars show the minimum and maximum values also the middle line shows the mean value, and the different letters above each Floating bars showed significantly different (*P* < 0.05) among the means, which are presented as mean ± SEM.

In the muscle of fish fed varying amounts of dietary PB, a substantial difference (*p* < 0.05) was seen in the levels of the analyzed gene expressions related to energy production. The up-regulated level of the *PGAM2* gene (2.283-fold increase) was observed in fish fed 2 g/kg PB in the diet (G3) in respect to the control and other supplemented groups, as indicated in Fig. 6.

### Liver and intestinal histomorphology

#### Liver histomorphology

**Fig. 7:**
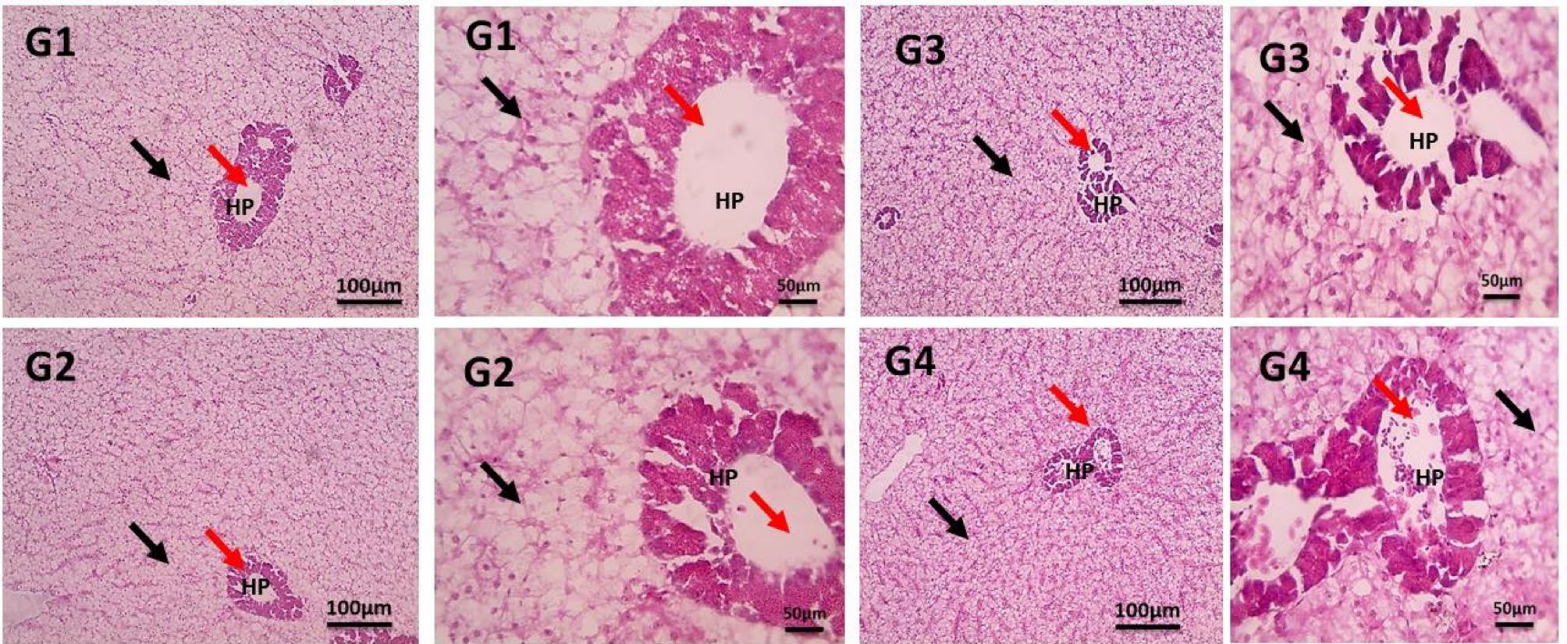
Microscopic pictures of H&E-stained liver sections showing normal hepatocytes (black arrows), hepatopancreas (HP) and blood vessels (red arrows). Low magnification X: 100 bar 100 and high magnification X: 400 bar 50. The control group, G1, received a basal diet, while G2, G3, and G4 received diets supplemented with 1 g, 2 g, and 4 g of PB powder per kilogram of feed, respectively for 90 days.

Figure 7 shows the results of varying food intake doses of PB on the liver histomorphology of examined Nile tilapia. All groups of Nile tilapia fed PB-supplemented diets exhibited normal liver tissue, with the group fed 2 g PB/kg (G3) showing the most typical liver structure, characterized by polygonal hepatocytes with prominent nuclei and normal blood capillaries. Additionally, exocrine pancreatic acini were well-organized with a large lumen in all liver tissues. In contrast, the normal liver architecture in the control group (G1) was slightly disorganized, with some cell hypertrophy.

#### Intestinal histomorphology

**Fig. 8:**
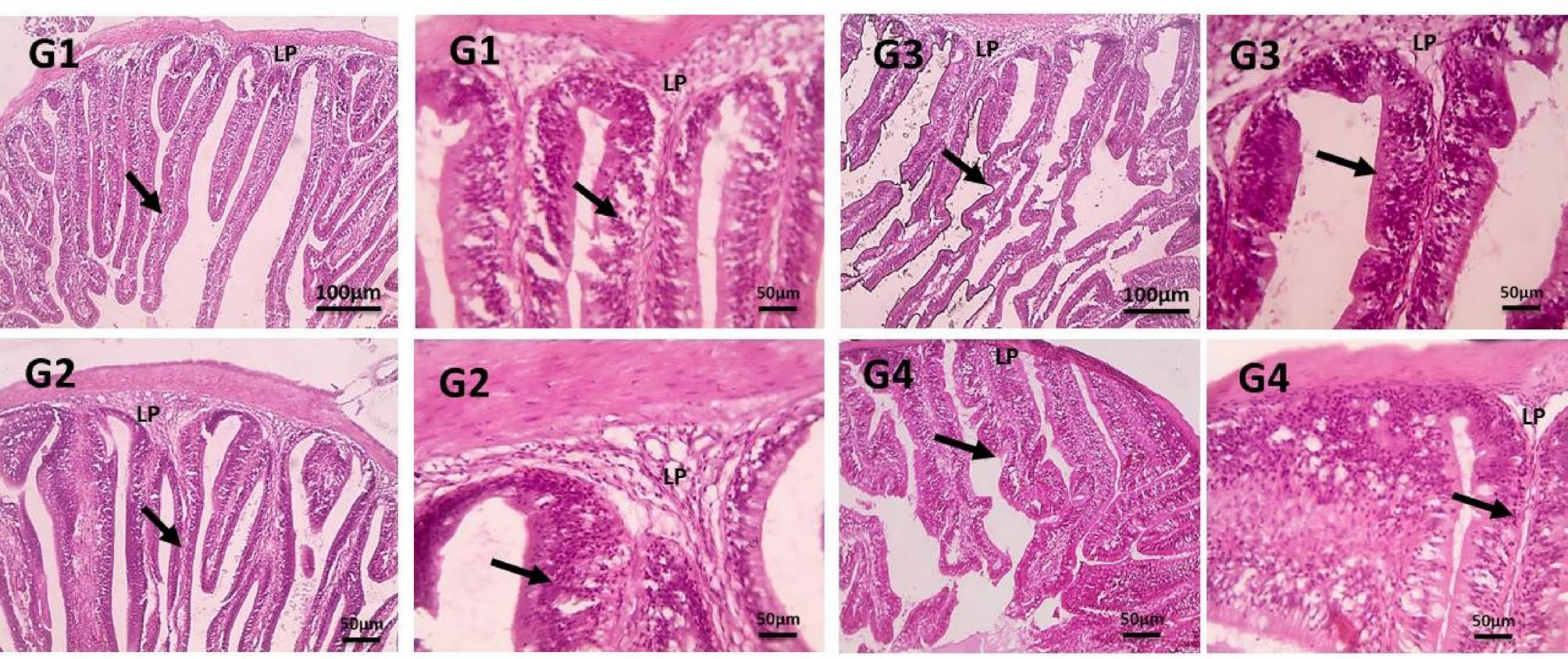
Microscopic pictures of H&E-stained intestine sections showing normal villi (black arrows), normal lamina propria (LP) with no evidence of any structural damage. Low magnification X: 100 bar 100 and high magnification X: 400 bar 50. The control group, G1, received a basal diet, while G2, G3, and G4 received diets supplemented with 1 g, 2 g, and 4 g of PB powder per kilogram of feed, respectively for 90 days.

The examined fish intestine sections show how all groups of Nile tilapia exhibited normal villi and lamina propria, as shown in Fig. 8. Moreover, the villi length and width in the intestinal tract of group G3 significantly increased compared to the other groups. Additionally, the lamina propria, a thin layer of connective tissue that lies beneath the epithelial lining of the intestine, was markedly thicker in group G3 compared to the other groups.

### Water quality

Table 6 explains no significant effect of diet supplement with PB (*p* > 0.05) on physical and chemical parameters of water quality. Temperature (°C), dissolved oxygen (mg/L), pH, NH3 (mg/L), and nitrite (mg/L) were within the normal level in the fish that received PB as well as the control group (G1).

**Table 6:** Water quality parameters (mean ± SE) of fish fed experimental diets for 90 days.

Items	G1	G2	G3	G4	*P*-value
Temperature (°C)	31.00 ± 0.377	29.05 ± 0.782	29.32 ± 0.463	30.38 ± 0.462	0.0649
Dissolved oxygen (mg/L)	8.000 ± 0.107	8.157 ± 0.073	7.857 ± 0.072	7.971 ± 0.057	0.0881
pH	8.067 ± 0.225	7.883 ± 0.214	7.785 ± 0.172	7.783 ± 0.194	0.0792
NH3 (mg/L)	0.217 ± 0.054	0.200 ± 0.052	0.217 ± 0.048	0.150 ± 0.034	0.7271
Nitrite (mg/L)	0.023 ± 0.007	0.018 ± 0.008	0.012 ± 0.008	0.008 ± 0.003	0.3927

Means within the same row lack common superscripts are significantly different at *P* < 0.05.

Dissolved oxygen (DO), unionized ammonia (NH_3_).

## Discussions

Microalgae technology has emerged as a promising technique to improve aquaculture system performance. Microalgae are rich in nutrient sources that can feed a range of farmed fish at various phases of development^44^. Many species of microalgae have been studied in fish feeding^45–47^ but there are still hundreds more that we know very little about in terms of their nutritional potential. *Pediastrum boryanum* (PB) is one of those species that could be really promising. The present study provides compelling evidence that PB is a viable feed ingredient for Nile tilapia (*Oreochromis niloticus*). Different doses of PB (0, 1, 2, and 4 g/kg) showed significant variations in growth performance, feed utilization, immune-physiological response, and transcriptome analysis.

Dietary supplementation with PB significantly improved the growth performance parameter, feed utilization, and survival rate of Nile tilapia also; a moderate concentration level of PB supplement 2 g/kg (G3) revealed an enhanced growth performance, feed utilization, lower FCR, and higher SGR in respect to other diets. The current experiment’s outcomes are consistent with previous studies on other microalgae like spirulina and chlorella that improved their feed intake, FCR and SGR^48,49^. Moreover, Nile tilapia that fed on PB extract supplements showed improvement in their growth performance^50^. Furthermore, fairy shrimp fed with PB had the longest body length (11.5 mm) during the first five days, followed by those fed dried chlorella (11.2 mm) and then the control group. Additionally, fairy shrimp fed with PB had significantly higher survival rates (96.6%) than other treatments according to^51^. These findings could be attributed to the high amount of protein (crude protein equal to 47.56%) in PB microalgae and diet supplemented with 2 g/kg (G3) recorded high protein level (31.58%) and low fiber (7.12%) as reported in the feed analysis of the current research. Fiber reduces the availability of nutrients and energy from other foods by interfering with their digestion and absorption^36,52^. On the other hand, carbon dioxide (CO_2_) and solar energy are needed for living PB algae, and a few necessary nutrients to create bio-oil more quickly than conventional oilseed crops like olive, sunflower, and soybean, which require a wide range of inputs, including land, water, and fertilizers^53^. That makes PB powder with high nutritional values. Therefore, using PB as a feed supplement can enhance Nile tilapia’s growth performance and survival rate.

The blood parameters of experimental meals administered to Nile tilapia were within the normal ranges observed in healthy fish^54^. The hematological profile of Nile tilapia fed a diet containing PB at a rate of 2–4 g/kg exhibited higher levels of RBCs, Hb, lymphocytes, and neutrophils. The tilapia fed PB supplemented diets exhibited normal blood biochemical values. Furthermore, the results showed that PB supplementation improved serum TP, albumin, globulins, triglycerides, creatinine, and ALT levels. These results match with^25^. According to^25^ who examined the effects of spirulina platensis (SP) and sodium butyrate (SB) supplements in the diet of *Oreochromis niloticus* on their blood parameters and state of health, feeding fish total protein was highest in SB plus SP, while AST, ALP, and lipid levels were the lowest in this combination. Moreover^55^, found that PB had no negative impact on the blood parameters when administered orally to mice. These findings may be related to the pharmacological potential of PB in improving health conditions. Thus, PB supplementation in the diet of Nile tilapia improved blood parameters, including hematological profile and biochemical values and maintained the blood health of the examined fish.

The current study’s findings show that fish fed on PB exhibited higher immune and antioxidant parameters, particularly (G3), in which fish fed at a rate of 2 g/Kg recorded higher levels of immunity and antioxidant parameters than fish fed in other groups. According to^56^ PB microalga have several advantages for immunity and antioxidant levels. Who explains why a rat model of carrageenan-induced paw edema had reduced cytokine levels and anti-inflammatory qualities. Moreover, our findings are similar to those of^57^who noticed that the health promotion of Nile tilapia could be enhanced in response to feeding with a low dose of *Spirulina platensis* and betaine. This outcome could be attributed to the advantageous chemicals found in PB. It generates high-value secondary metabolites, including phenolic compounds^28^ vitamins^58^ polyunsaturated fatty acids^59^ and carotenoids^60^. Most of these substances have shown strong antioxidant properties. Overall, PB has the potential to enhance immune function and support antioxidant defenses in Nile tilapia.

There is up-regulation of *GH*, *IGF-1*,* SOD*,* Catalase*,* IL6*,* IL8*, and *PGAM2* genes in fish fed PB supplement diets, with the most significant high variables found in the 2 g/kg (G3). This study found that feeding PB to fish upregulates *GH* and *IGF-1* genes in the liver. Similar findings have been observed in other studies, which have demonstrated that dietary interventions, such as supplementation with *Ulva fasciata* extract^14^different dietary lipid levels^61^ and fructooligosaccharide^62^. Our findings of upregulated superoxide dismutase (SOD) and catalase genes in the liver of fish that feed PB are supported by previous research by^63^ who reported significantly upregulated SOD and catalase genes in rainbow trout fed a diet containing 10% *Spirulina platensis* microalgae. Additionally, our findings of upregulated *IL-6* and *IL-8* genes in the spleen of fish that feed on PB are supported by^64^ who found that the *IL-6* and *IL-8* genes, which are critical immune system mediators that facilitate the fish’s response to bacterial infections, were expressed more highly in all groups of European seabass that were fed spirulina. Finally, our findings of upregulated *PGAM2* genes in the muscle of fish that feed on Pediastrum are supported by^65^ who confirmed that high expression of the phosphoglycerate mutase 2 (PGAM2) gene in pufferfish exposed to acute hypoxia which suggests that this gene plays a vital role in energy coordination. The observed findings could be due to the fact that the administration of *Pediastrum boryanum* has no negative impact on DNA according to^21^. On the other hand, the most comprehensive physicochemical characterization of the elemental composition of PB has revealed that it contains the following elements: carbon, oxygen, aluminum, nitrogen, sulfur, phosphorus, and sodium that recorded in^66^. SO that dietary supplementation of PB can upregulate the expression of genes involved in growth, antioxidant capacity, immune function, and energy coordination in fish.

In addition to vitamins and minerals, the liver also has enzymes that aid in the breakdown of fats and carbohydrates. The liver also stores important nutrients such as blood sugar, lipids, vitamin A, and vitamin D, as well as aiding in the prevention of blood clotting in fish bodies^67^. This study revealed that Nile tilapia fed PB showed normal liver tissue, with the 2 g/kg group (G3) showing a typical liver structure with polygonal hepatocytes and normal blood capillaries. In line with these findings, fish regularly fed once or twice a day had enhanced liver organelle structure that showed normal hepatocytes, central vein, and hepatopancreas^68^. Furthermore^69^, conducted a microscopic examination of the liver of Nile tilapia that fed on probiotic *Pediococcus acidilactici*, the histological structure was normal and the central vein was surrounded by polyhedral vacuolated hepatocytes. PB can help to improve liver detoxification. This is because it contains antioxidants^21^ which help to protect the liver from damage caused by free radicals. So dietary supplementation of Nile tilapia with PB showed normal liver morphology.

The intestine plays a critical role in nutrient digestion and absorption, and its function is enhanced by improved intestinal structure, as demonstrated by the increased intestinal villus height that results in a larger nutrient absorption surface area^70^. These results are attributed to the beneficial effects of PB on intestinal health attributed to its essential fatty acid content^59^ which helps to maintain the integrity of the intestinal epithelium; its prebiotic content^71^ which promotes the growth of helpful bacterium in the digestive system; and its anti-inflammatory properties^72^which can help to reduce intestinal inflammation. Our study showed Nile tilapia had typical villi and lamina propria; however, in contrast to other groups, group G3 exhibited noticeably longer and broader villi and thicker lamina propria under the intestinal lining. Concurrent with these results^25^, explains that intestinal histomorphology of Nile tilapia showed a significant increase in the intestinal villi length, villi surface area, and goblet cell count and diets supplemented with sodium butyrate and *Spirulina platensis* compared to the control. Furthermore, it was demonstrated in^73^ that Nile tilapia, given supplement diets containing 0.5–1% Rhodotorula mucilaginosa, had considerably longer villi than the control group. Overall, the evidence suggests that PB can help to improve intestinal morphometry and overall gut health in Nile tilapia.

Throughout the trial period, all tanks’ water temperatures (°C), dissolved oxygen (mg/L), pH, NH3 (mg/L), and nitrite (mg/L) stayed consistent and within the optimal range for the cultivation of Nile tilapia^74,75^. The study’s findings demonstrated that there were no appreciable variations in the physical and chemical characteristics of the water quality across all groups, whether or not they were given PB supplements. Perhaps as a result of the microalgae PB material, which, as noted in^76^ can be employed as an efficient sorbent to remove lead, cadmium, and copper from contaminated wastewater at low pH levels. In line with these findings, using a combination of beneficial bacteria had no influence on the Nile tilapia’s water quality^77^. Moreover^78^, Who feed Nile tilapia with *Nannochloropsis sp.* and *Tetraselmis sp.* microalgae show no significant change in physical parameters of water quality. All things considered, PB has no negative effects on water quality and can even assist Nile tilapia to thrive in it.

## Conclusion

In conclusion, supplementation of the diet with *Pediastrum boryanum* (PB) microalgae at a level of 2 g/kg improved growth performance, as evidenced by elevated levels of red blood cells (RBCs) and neutrophils. Furthermore, G3 demonstrated the highest upregulation of genes associated with growth, antioxidant capacity, and immune response. These findings underscore the potential of PB supplementation in enhancing various physiological aspects of Nile tilapia. Future investigations of PB supplementation should be on some heavy metal where Nile tilapia lives.

## Data Availability

All relevant data are available from the corresponding author upon request.

## References

[1] FAO. The state of food and agriculture making agrifood systems more resilient to shocks and stresses. *Rome FAO*. 10.4060/cb4476en (2021).

[2] Pradeepkiran, J. A. Aquaculture role in global food security with nutritional value: A review. *Transl. Anim. Sci.* **3**, 903–910 (2019). 32704855 10.1093/tas/txz012 PMC7200472

[3] Amuneke, K. E. et al. Impact of temperature manipulations on growth performance, body composition, and selected genes of Koi carp (Cyprinus Carpio koi). *Fishes* **10**(95). 10.3390/fishes10030095 (2025).

[4] Prabu, E., Rajagopalsamy, C., Ahilan, B., Jeevagan, I. & Renuhadevi, M. Tilapia–an excellent candidate species for world aquaculture: a review. *Annual Res. Rev. Biol.* **31**, 1–14 (2019).

[5] Ahsan, M. et al. Impacts of inclusion of column feeder Rohu (*Labeo rohita*) at different stocking densities on growth, production and environment in freshwater prawn-carp-mola polyculture system. *Int. J. Biol. Res.* **1**, 48–54 (2013).

[6] El-Sayed, A. F. M. & Fitzsimmons, K. From Africa to the world—the journey of Nile tilapia. *Reviews Aquaculture*. **15**, 6–21 (2023).

[7] FAO. The State of Food Security and Nutrition in the World: 2022: Repurposing Food and Agricultural Policies to Make Healthy Diets More Affordable (FAO, 2022).

[8] Norman, R., Crumlish, M. & Stetkiewicz, S. The importance of fisheries and aquaculture production for nutrition and food security. *Revue scientifique et technique (International Office of Epizootics)* **38**, 395–407 (2019). 10.20506/rst.38.2.2994 31866686

[9] Khalfallah, M., Mahmoud, H. H., Fahim, R. M. & Pauly, D. Once upon a century, the Egyptian mediterranean fisheries (1920–2019), as affected by ‘fishing down’and climate change. *Ocean. Coastal. Manag.* **245**, 106831 (2023).

[10] Walakira, J. K. et al. Scaling aquaculture for food security and employment in Africa–Insights from Egypt, Kenya and Nigeria. (2023).

[11] Ahmed, A. B. A., Adel, M., Karimi, P. & Peidayesh, M. Pharmaceutical, cosmeceutical, and traditional applications of marine carbohydrates. *Adv. Food Nutr. Res.* **73**, 197–220 (2014). 25300548 10.1016/B978-0-12-800268-1.00010-X

[12] Abdella, B., Abozahra, N. A., Shokrak, N. M. & Mohamed, R. A. Whole spectrum of *Aeromonas hydrophila* virulence determinants and the identification of novel SNPs using comparative pathogenomics. *Sci. Rep.* **13**, 7712 (2023). 37173388 10.1038/s41598-023-34887-1 PMC10182093

[13] El Saidy, N. R. et al. Wastewater remediation of heavy metals and pesticides using rice straw and/or zeolite as bioadsorbents and assessment of treated wastewater reuse in the culture of Nile tilapia (*Oreochromis niloticus*). *Environ. Monit. Assess.* **192**, 1–21 (2020). 10.1007/s10661-020-08760-x 33230706

[14] Abo-Raya, M. H. et al. Assessment of growth‐related parameters and immune‐biochemical profile of Nile tilapia (*Oreochromis niloticus*) fed dietary ulva fasciata extract. *Aquac. Res.* **52**, 3233–3246 (2021).

[15] Abozahra, N. A., Albalawi, A., Althobaiti, N., Mohamed, R. & Diab, A. Effects of pomegranate (*Punica granatum*) Peel methanolic extract dietary supplementation on *Oreochromis niloticus* performance, blood health, intestine morphometry and immunity. *Egypt. J. Vet.Sci.* **54**, 221–236 (2023).

[16] Elkaradawy, A., Abdel-Rahim, M. M. & Mohamed, R. A. Quillaja saponaria and/or linseed oil improved growth performance, water quality, welfare profile and immune‐oxidative status of Nile tilapia, *Oreochromis niloticus* fingerlings. *Aquac. Res.* **53**, 576–589 (2022).

[17] El-Tawil, N. E. Effects of green seaweeds (*Ulva* sp.) as feed supplements in red tilapia (*Oreochromis* sp.) diet on growth performance, feed utilization and body composition. *J. Arab. Aquaculture Soc.* **5**, 179–194 (2010).

[18] Khaligh, S. F. & Asoodeh, A. Recent advances in the bio-application of microalgae-derived biochemical metabolites and development trends of photobioreactor-based culture systems. *3 Biotech* **12**, 260 (2022). 36072963 10.1007/s13205-022-03327-8 PMC9441132

[19] Selvaraj, S., Bains, A., Sharma, M., Chawla, P. & Sridhar, K. Freshwater edible algae polysaccharides: A recent overview of novel extraction technologies, characterization, and future food applications. *J. Polym. Environ.* 1–25 (2023).

[20] Kothari, R. et al. Potential avenue of genetic engineered algal derived bioactive compounds: influencing parameters, challenges and future prospects. *Phytochem. Rev.* 1–34 (2023).

[21] Silva, M. G. C. et al. Anti-inflammatory and antioxidant effects of the microalga Pediastrum boryanum in Carrageenan-Induced rat paw edema. *Braz. Arch. Biol. Technol.* **64** (2022).

[22] Hussain, A., Aslam, B., Muhammad, F. & Faisal, M. N. In vitro antioxidant activity and in vivo anti-inflammatory effect of *Ricinus communis* (L.) and *Withania somnifera* (L.) hydroalcoholic extracts in rats. *Braz. Arch. Biol. Technol.* **64** (2022).

[23] Renuka, B. & Borse, B. Delivery of Probiotics in the Food Industries. *Novel Processing Methods for Plant-Based Health Foods: Extraction, Encapsulation, and Health Benefits of Bioactive Compounds*, 285 (2023).

[24] Elshafey, A. E., Khalafalla, M. M., Zaid, A. A. A., Mohamed, R. A. & Abdel-Rahim, M. M. Source diversity of artemia enrichment boosts goldfish (*Carassius auratus*) performance, β-carotene content, pigmentation, immune-physiological and transcriptomic responses. *Sci. Rep.* **13**, 21801 (2023). 38065998 10.1038/s41598-023-48621-4 PMC10709595

[25] Shalata, H. A. et al. Synergistic effects of dietary sodium butyrate and *Spirulina platensis* on growth performance, carcass composition, blood health, and intestinal histomorphology of Nile tilapia (*Oreochromis niloticus*). *Aquaculture Rep.* **19**, 100637. 10.1016/j.aqrep.2021.100637 (2021).

[26] Wu, J. et al. Bioactive substances and potentiality of marine microalgae. *Food Sci. Nutr.* **9**, 5279–5292 (2021). 34532034 10.1002/fsn3.2471 PMC8441504

[27] Mobin, S. M., Chowdhury, H. & Alam, F. Commercially important bioproducts from microalgae and their current applications–A review. *Energy Proc.* **160**, 752–760 (2019).

[28] Corrêa da Silva, M. G. et al. Phenolic compounds and antioxidant capacity of *Pediastrum boryanum* (Chlorococcales) biomass. *Int. J. Environ. Health Res.* **32**, 168–180 (2022). 32200653 10.1080/09603123.2020.1744113

[29] Jacinto, G. S. S. et al. Biotechnological investigation of Pediastrum boryanum and desmodesmus subspicatus microalgae species for a potential application in bioenergy. *Algal Res.* 103266 (2023).

[30] AOAC. Association of official analytical chemists. *Official Methods Analysis* 15th edn (2003).

[31] ISO. *Animal Feeding Stuffs, Animal Products, and Faeces or Urine: Determination of Gross Calorific Value: Bomb Calorimeter Method* (International Organization for Standardization, 1998).

[32] Elkadom, E. M., El-Kader, A., Bakr, M. F., Abozeid, B. A., Mohamed, R. A. & A. M. & Impacts of various single and mixed colors of monochromatic LED light on growth, behavior, immune-physiological parameters, and liver and brain histology of Nile tilapia fingerlings. *Aquaculture* **577**, 740007 (2023).

[33] Bittencourt, N. L. R., Molinari, L. M., de Oliveira, D., de Abreu Filho, B. A. & Dias Filho, B. P. Haematological and biochemical values for Nile tilapia Oreochromis niloticus cultured in semi-intensive system. *Hemoglobin (g/dl)* **10**, 6.58–15.98 (2003).

[34] Anderson, D. & Siwicki, A. Basic hematology and serology for fish health programs (1995).

[35] Palti, Y. et al. Comparative study of biochemical and non-specific immunological parameters in two tilapia species (Oreochromis aureus and O. mossambicus). *Israeli J. Aquaculture Bamidgeh*. **51**, 148–156 (1999).

[36] Vasconcelos, T. S. et al. Evaluation of pineapple byproduct at increasing levels in heavy finishing pigs feeding. *Anim. Feed Sci. Technol.* **269**, 114664 (2020).

[37] Doumas, B. T., Watson, W. A. & Biggs, H. G. Albumin standards and the measurement of serum albumin with bromcresol green. *Clin. Chim. Acta* **31**, 87–96 (1971). 5544065 10.1016/0009-8981(71)90365-2

[38] Heinegård, D. & Tiderström, G. Determination of serum creatinine by a direct colorimetric method. *Clin. Chim. Acta* **43**, 305–310 (1973). 4690902 10.1016/0009-8981(73)90466-x

[39] Demers, N. E. & Bayne, C. J. The immediate effects of stress on hormones and plasma lysozyme in rainbow trout. *Dev. Comp. Immunol.* **21**, 363–373 (1997). 9303274 10.1016/s0145-305x(97)00009-8

[40] Hao, K. et al. Functional identification of an FMRFamide-related peptide gene on diapause induction of the migratory locust, *Locusta migratoria* L. *Genomics* **112**, 1821–1828 (2020). 31669703 10.1016/j.ygeno.2019.10.015

[41] Pfaffl, M. W. A new mathematical model for relative quantification in real-time RT–PCR. *Nucleic Acids Res.* **29**, e45–e45 (2001). 11328886 10.1093/nar/29.9.e45 PMC55695

[42] Suvarna, K. S., Layton, C. & Bancroft, J. D. *Bancroft’s theory and practice of histological techniques* (Elsevier Health Sciences, 2018).

[43] Moopam, R. Manual of oceanographic observations and pollutant analysis methods. *ROPME Kuwait*. **1**, 122–133 (1999).

[44] Nagappan, S. et al. Potential of microalgae as a sustainable feed ingredient for aquaculture. *J. Biotechnol.* **341**, 1–20 (2021). 34534593 10.1016/j.jbiotec.2021.09.003

[45] Altmann, B. A. & Rosenau, S. Spirulina as animal feed: opportunities and challenges. *Foods* **11**, 965 (2022). 35407052 10.3390/foods11070965 PMC8997485

[46] Ahmad, A., Hassan, W., Banat, F. & S. & An overview of microalgae biomass as a sustainable aquaculture feed ingredient: Food security and circular economy. *Bioengineered* **13**, 9521–9547 (2022). 35387561 10.1080/21655979.2022.2061148 PMC9161971

[47] Tham, P. E. et al. Insights of microalgae-based aquaculture feed: A review on circular bioeconomy and perspectives. *Algal Res.* 103186 (2023).

[48] AlMulhim, N. M., Virk, P., Abdelwarith, A. A. & AlKhulaifi, F. M. Effect of incorporation of *Spirulina platensis* into fish diets, on growth performance and biochemical composition of Nile tilapia, Oreochromis niloticus. *The Egypt. J. Aquat. Research* (2023).

[49] Abdel-Tawwab, M. et al. Dietary *Chlorella vulgaris* modulates the performance, antioxidant capacity, innate immunity, and disease resistance capability of Nile tilapia fingerlings fed on plant-based diets. *Anim. Feed Sci. Technol.* **283**, 115181 (2022).

[50] Al-Wakeel, A. H., Elbahnaswy, S., Risha, E. & Zahran, E. Dietary *Pediastrum boryanum* microalgal extract improves growth, enhances immunity, and regulates immune-related genes in Nile tilapia. *BMC Vet. Res.* **20**, 321. 10.1186/s12917-024-04155-z (2024). 39026262 10.1186/s12917-024-04155-z PMC11256681

[51] Plodsomboon, S. & Sanoamuang, L. Effects of *Pediastrum boryanum* and dried *Chlorella* as feeds on the growth performance and carotenoid content of the fairy shrimp *Branchinella thailandensis* (Branchiopoda, Anostraca). *Trop. Nat. History* **23**, 73–81 (2023).

[52] Lin, S. M., Shi, C. M., Mu, M. M., Chen, Y. J. & Luo, L. Effect of high dietary starch levels on growth, hepatic glucose metabolism, oxidative status and immune response of juvenile largemouth bass, *Micropterus salmoides*. *Fish Shellfish Immunol.* **78**, 121–126 (2018). 29684600 10.1016/j.fsi.2018.04.046

[53] Chinnasamy, S., Ramakrishnan, B., Bhatnagar, A. & Das, K. C. Biomass production potential of a wastewater alga *Chlorella vulgaris* ARC 1 under elevated levels of CO2 and temperature. *Int. J. Mol. Sci.* **10**, 518–532 (2009). 19333419 10.3390/ijms10020518 PMC2660655

[54] Mohamed, R. A., Aboulila, A. A., El-Kholya, S. Z. & Hamza, A. Evaluation of genetic polymorphism, genomic template stability, condition factor and hemato-biochemical parameters in response to slow reduction in water level during Nile tilapia (*Oreochromis niloticus*) harvest. *Thai J. Vet. Med.* **47**, 435–448 (2017).

[55] Fonseca, A. F. et al. Evaluation of acute toxicity of the microalgae *Pediastrum boryanum*. *Vittalle* **28**, 90–102 (2016).

[56] Silva, M. G. C. et al. Anti-inflammatory and antioxidant effects of the microalga *Pediastrum boryanum* in Carrageenan-Induced rat paw edema. *Braz. Arch. Biol.Technol.* **64** (2021).

[57] Awad, L. Z. et al. Role of dietary Spirulina platensis and betaine supplementation on growth, hematological, serum biochemical parameters, antioxidant status, immune responses, and disease resistance in Nile tilapia. *Fish Shellfish Immunol.* **126**, 122–130 (2022). 35613669 10.1016/j.fsi.2022.05.040

[58] Sutkowy, M., Lenarczyk, J. & Kłosowski, G. Effect of culture medium on the growth of microscopic algae (Chlorophyceae) biomass showing biosorption potential: A case study pseudo *Pediastrum boryanum*. *Phycological Res.* **67**, 112–119 (2019).

[59] Blokker, P. et al. Chemical structure of Algaenans from the fresh water algae Tetraedron minimum, *Scenedesmus communis* and *Pediastrum boryanum*. *Org. Geochem.* **29**, 1453–1468 (1998).

[60] Tenorio, C., Ramírez, J. A. H., Ramos, L. F., Soto, A. R. & Vargas, J. Effects of ultraviolet radiation on production of photoprotective compounds in microalgae of the genus Pediastrum from high Andean areas of Peru. *J. Appl. Pharm. Sci.* **12**, 087–095 (2022).

[61] Fan, Z. et al. Excessive dietary lipid affecting growth performance, feed utilization, lipid deposition, and hepatopancreas lipometabolism of large-sized common carp (Cyprinus carpio). *Front. Nutr.* **8**, 694426 (2021). 34327208 10.3389/fnut.2021.694426 PMC8313730

[62] Jiang, D. et al. Protective effects of the fructooligosaccharide on the growth performance, biochemical indexes, and intestinal morphology of blunt snout Bream (*Megalobrama amblycephala*) infected by *Aeromonas hydrophila*. *Fish Physiol. Biochem.* **49**, 139–153 (2023). 36538149 10.1007/s10695-022-01162-5

[63] Teimouri, M., Yeganeh, S., Mianji, G. R., Najafi, M. & Mahjoub, S. The effect of *Spirulina platensis* meal on antioxidant gene expression, total antioxidant capacity, and lipid peroxidation of rainbow trout (*Oncorhynchus mykiss*). *Fish Physiol. Biochem.* **45**, 977–986 (2019). 30648194 10.1007/s10695-019-0608-3

[64] Güroy, B. et al. Effect of dietary spirulina (*Arthrospira platensis*) on the growth performance, immune-related gene expression and resistance to *Vibrio anguillarum* in European Seabass (*Dicentrarchus labrax*). *Aquac. Res.* **53**, 2263–2274 (2022).

[65] Li, X. et al. The improved energy metabolism and blood oxygen-carrying capacity for pufferfish, *Takifugu fasciatus*, against acute hypoxia under the regulation of oxygen sensors. *Fish Physiol. Biochem.* **45**, 323–340 (2019). 30225749 10.1007/s10695-018-0565-2

[66] Jacinto, G. S. S. et al. Biotechnological investigation of *Pediastrum boryanum* and desmodesmus subspicatus microalgae species for a potential application in bioenergy. *Algal Res.* **75**, 103266. 10.1016/j.algal.2023.103266 (2023).

[67] Bruslé, J. & i Anadon, G. G. *Fish Morphology* 77–93 (Routledge, 2017).

[68] El-Araby, D. A., Amer, S. A. & Khalil, A. A. Effect of different feeding regimes on the growth performance, antioxidant activity, and health of Nile tilapia, *Oreochromis niloticus*. *Aquaculture* **528**, 735572. 10.1016/j.aquaculture.2020.735572 (2020).

[69] El-Haroun, E. et al. Dietary inclusion of *Pediococcus Acidilactici*probiotic promoted the growth indices, Hemato-Biochemical indices, enzymatic profile, intestinal and liver histomorphology, and resistance of Nile tilapia against *Aspergillus flavus*. *Pediococcus Acidilactici* (2023).

[70] Zhu, H., Liu, H., Yan, J., Wang, R. & Liu, L. Effect of yeast polysaccharide on some hematologic parameter and gut morphology in channel catfish (*Ictalurus punctatus*). *Fish Physiol. Biochem.* **38**, 1441–1447 (2012). 22437370 10.1007/s10695-012-9631-3

[71] Santiago-Díaz, P., Rivero, A., Rico, M. & Gómez-Pinchetti, J. L. Characterization of novel selected microalgae for antioxidant activity and polyphenols, amino acids, and carbohydrates. *Mar. Drugs* **20**, 40 (2021). 35049895 10.3390/md20010040 PMC8777807

[72] Nwankwo, N. E., Aham, E. C., Ezenabor, E. H. & Chidozie, D. M. Anti-inflammatory and antioxidant activities of ethylacetate fraction of *Sida linifolia* L.(Malvaceae). *Int. J. Plant. Based Pharm.* **3**, 200–209 (2023).

[73] Chen, X. Q. et al. Effects of dietary hydrolyzed yeast (*Rhodotorula mucilaginosa*) on growth performance, immune response, antioxidant capacity and histomorphology of juvenile Nile tilapia (*Oreochromis niloticus*). *Fish Shellfish Immunol.* **90**, 30–39 (2019). 31004799 10.1016/j.fsi.2019.03.068

[74] Tran-Duy, A., van Dam, A. A. & Schrama, J. W. Feed intake, growth and metabolism of Nile tilapia (Oreochromis niloticus) in relation to dissolved oxygen concentration. *Aquac. Res.* **43**, 730–744 (2012).

[75] Santos, V. B. & d., Mareco, E. A. Growth curves of Nile tilapia (*Oreochromis niloticus*) strains cultivated at different temperatures. *Acta Sci. Anim. Sci.* **35**, 235–242 (2013).

[76] Joseph, J. et al. A comparative study of modified and unmodified algae (*Pediastrum boryanum*) for removal of lead, cadmium and copper in contaminated water. *Water Qual.* **11**, 245–266 (2017).

[77] Khalafalla, M. M., Ibrahim, S. A., Zayed, M. M., Awad, M. N. & Mohamed, R. A. Effect of a dietary mixture of beneficial bacteria on growth performance, health condition, chemical composition, and water quality of Nile tilapia, *Oreochromis niloticus* fingerlings. *J. Aquat. Food Prod. Technol.* **29**, 823–835 (2020).

[78] Amira, K. I. et al. Data on growth, survivability, water quality and hemato-biochemical indices of Nile tilapia (*Oreochromis niloticus*) fry fed with selected marine microalgae. *Data Brief.* **38**, 107422. 10.1016/j.dib.2021.107422 (2021). 34632018 10.1016/j.dib.2021.107422 PMC8488251

